# *Clostridium botulinum* type C, D, C/D, and D/C: An update

**DOI:** 10.3389/fmicb.2022.1099184

**Published:** 2023-01-05

**Authors:** François Meurens, Frédéric Carlin, Michel Federighi, Maria-Eleni Filippitzi, Matthieu Fournier, Philippe Fravalo, Jean-Pierre Ganière, Lionel Grisot, Laurent Guillier, Didier Hilaire, Pauline Kooh, Sophie Le Bouquin-Leneveu, Caroline Le Maréchal, Christelle Mazuet, Hervé Morvan, Karine Petit, Jean-Pierre Vaillancourt, Cédric Woudstra

**Affiliations:** ^1^INRAE, Oniris, BIOEPAR, Nantes, France; ^2^Department of Veterinary Microbiology and Immunology, Western College of Veterinary Medicine, University of Saskatchewan, Saskatoon, SK, Canada; ^3^INRAE, Avignon Université, UMR SQPOV, Avignon, France; ^4^SECALIM, INRAE, Oniris, Nantes, France; ^5^Laboratory of Animal Health Economics, Aristotle University of Thessaloniki, Thessaloniki, Greece; ^6^Univ Rouen Normandie, Univ Caen Normandie, CNRS, M2C, UMR 6143, Rouen, France; ^7^Chaire Agroalimentaire du Cnam, Conservatoire des Arts et Métiers, EPN7, Ploufragan, France; ^8^Retired, Brittany, France; ^9^Clinique des Tourbières, Frasne, France; ^10^Risk Assessment Department, ANSES, Maisons-Alfort, France; ^11^DGA Maîtrise NRBC, Vert-le-Petit, France; ^12^Hygiene and Quality of Poultry and Pig Products Unit, ANSES, French Agency for Food, Environmental and Occupational Health Safety, Ploufragan, France; ^13^Institut Pasteur, Université Paris Cité, CNR Bactéries anaérobies et Botulisme, Paris, France; ^14^Department of Clinical Sciences, Faculty of Veterinary Medicine, Université de Montréal, Saint-Hyacinthe, QC, Canada; ^15^Department of Veterinary and Animal Sciences, University of Copenhagen, Frederiksberg C, Denmark

**Keywords:** *Clostridium*, zoonosis, cattle, poultry, toxins, botulism

## Abstract

*Clostridium botulinum* is the main causative agent of botulism, a neurological disease encountered in humans as well as animals. Nine types of botulinum neurotoxins (BoNTs) have been described so far. Amongst these “toxinotypes,” the A, the B and E are the most frequently encountered in humans while the C, D, C/D and D/C are mostly affecting domestic and wild birds as well as cattle. In France for instance, many cases and outbreaks are reported in these animal species every year. However, underestimation is very likely at least for avifauna species where the detection of dead animals can be challenging. Knowledge about BoNTs C, D, C/D, and D/C and the diseases they cause in animals and humans is still scarce and unclear. Specifically, the potential role of animal botulism outbreaks in cattle and poultry as a source of human illness needs to be further assessed. In this narrative review, we present the current knowledge about toxinotypes C, D, C/D, and D/C in cattle and poultry with, amongst various other aspects, their epidemiological cycles. We also discuss the zoonotic potential of these toxinotypes and some possible ways of risk mitigation. An adapted and effective management of botulism outbreaks in livestock also requires a better understanding of these less common and known toxinotypes.

## 1. Introduction

Botulism is a human and animal neurological disease caused by the action of bacterial neurotoxins produced by Gram-positive bacteria of the genus *Clostridium* (Hodowanec and Bleck, 2015). Typically, a flaccid paralysis, that can lead in worst cases to respiratory paralysis and heart failure causing death, is observed (Torrens, 1998; Hodowanec and Bleck, 2015; Le Maréchal et al., 2016b). Nine types of botulinum neurotoxins (BoNTs) or toxinotypes have been described (Peck et al., 2017; Doxey et al., 2018). While human botulism is mostly associated with types A, B and E and less frequently with types F, different types are associated with animal botulism, mainly in birds (wild and domestic) and cattle (Le Maréchal et al., 2016b). In birds, the toxinotypes involved are the mosaic C/D (majority), mosaic D/C, D and C (rarely) and exceptionally type E. On a world-wide basis, avian botulism is the most significant disease of waterbirds. Outbreaks in wild birds have been reported worldwide, except in Antarctica, with losses that have sometimes exceeded 50,000 dead birds (Rocke, 2006). In cattle, the toxinotypes involved are the mosaic D/C type (majority), C, mosaic C/D and rarely D. In France, the incidence over the last 10 years is on average about 10 to 30 outbreaks per year depending on the animal species considered (Le Maréchal et al., 2016b; Le Bouquin et al., 2022). However, this is probably an underestimation: in the avifauna (wild and non-captive) in particular, detection and reporting of dead birds is much less systematic than it could be for dead mammals. The literature about BoNT/C, D, C/D, and D/C mosaics (see Section 1.2) is definitely less abundant than for other toxinotypes. More specifically, the potential role of the outbreaks in cattle and poultry as a source of human contamination and ultimately of human botulism needs to be addressed. In this narrative review, we update the general knowledge about C, D, and mosaics C/D and D/C based on the situation in France, but we present this knowledge enhanced with a special focus on their zoonotic potential and related health consequences from a One Health perspective.

### 1.1. *Clostridium botulinum* – The bacteria

*Clostridium botulinum* consists of a group of Gram-positive, rod shaped, spore forming anaerobic bacteria (1.6–22 μm long and 0.5–2 μm wide) of the genus *Clostridium* whose common feature is the ability to synthesize a protein toxin called BoNT (Cato et al., 1986; Le Maréchal et al., 2016b; Poulain and Popoff, 2019), responsible for botulism, a severe neurological condition that causes flaccid paralysis. The genus *Clostridium* consists of about 200 species, of which about fifteen can synthesize toxins that cause disease in humans or animals (Poulain and Popoff, 2019). *C. botulinum* presents a great genetic diversity and is currently classified into three groups according to their biochemical, noteworthy proteolytic, characteristics (Poulain and Popoff, 2019). Thus, group I is proteolytic while groups II and III are non-proteolytic. Other species of the genus *Clostridium* can also produce BoNTs and constitute groups IV (so called *C. argentinense*), V (*C. baratii*) and VI (*C. butyricum*) of BoNT producing Clostridia (Smith et al., 2018). Group IV is proteolytic while groups V and VI are non-proteolytic.

The bacteria, in their vegetative form, are peritrichous and motile bacteria (Hodowanec and Bleck, 2015; Le Maréchal et al., 2016b). The metabolism of *C. botulinum* is of the chemo-organotrophic type, the end products of metabolism being acetic, butyric and propionic acids. Clostridia have a strictly anaerobic respiratory type. Some strains can tolerate low oxygen in their growing environment. Because of their metabolic characteristics, *Clostridium* species including *C. botulinum* are involved in degradation of organic matter. The physiological role of BoNTs is still not known but, according to DasGupta (2006), their presence is definitely not essential for the survival and the growth of *C. botulinum* strains (DasGupta, 2006).

### 1.2. Toxins

**Figure 1:**
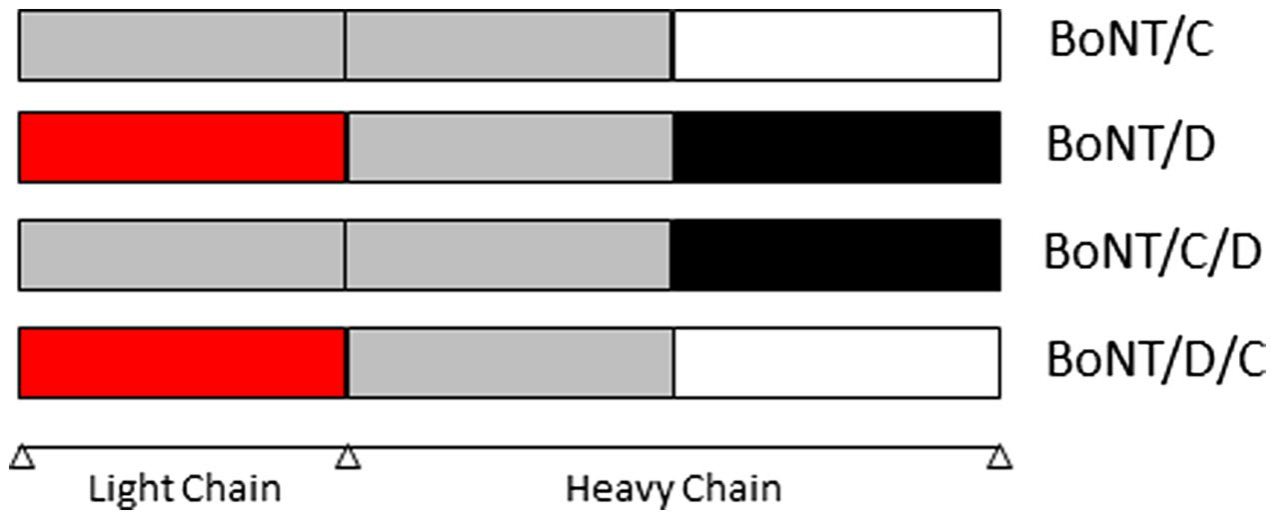
Structure of BoNT/C, /D, /CD et /DC (BoNT: *Botulinum* neurotoxin).

BoNTs are a heterogeneous family of proteins produced by *C. botulinum* as well as some strains of *Clostridium butyricum* and *Clostridium baratii* (Poulain and Popoff, 2019). BoNTs share a similar structure (DasGupta, 2006) and are synthetized as a single-chain polypeptide (approximately 150 kDa) that is cleaved by a protease into dichain proteins linked by a disulfide bond (Montecucco, 1986). The dichain protein is composed of a 50 kDa light chain (LC) with zinc-dependent protease activity and a 100 kDa heavy chain (HC) with an N-terminal translocation domain (Hn) and a C-terminal cell binding domain (Hc). BoNTs are divided into nine toxinotypes (A to H and X) based on neutralization of toxicity by specific antisera, using the mouse biological test and specific neutralizing antisera (Solomon and Lilly, 2001; Peck et al., 2017; Zhang et al., 2017b). BoNT genes have been sequenced from many strains and sequence comparisons have enabled the identification of sequence variations in each toxinotype. Thereby, BoNTs are divided into subtypes (Peck et al., 2017). There are eight subtypes identified for BoNT/A (A1 – A8), BoNT/B (B1 – B8) and BoNT/F (F1 – F8); twelve for BoNT/E (E1 – E12). No subtype has been identified for the other toxinotypes. However, there are two mosaic hybrid forms of BoNT/C and BoNT/D named BoNT/C/D and BoNT/D/C. BoNT/C/D is composed of the light chain of BoNT/C and the heavy chain of BoNT/D. BoNT/D/C is composed of the light chain of BoNT/D and the heavy chain of BoNT/C (Figure 1). BoNT/H is a BoNT/A and/F hybrid and might also been considered as BoNT/A/F (Barash and Arnon, 2014; Maslanka et al., 2016).

The consequence of sequence diversity in each toxinotype and subtype is not yet clear. We can speculate it might affect the properties of each BoNT including stability, receptor recognition, enzymatic activity, efficiency of entry into cells, and recognition by antibodies (identification and immunotherapy).

BoNTs are produced as protein complexes of different sizes (Schantz and Spero, 1967; Popoff, 2018). In the complex form, BoNTs are associated by non-covalent bounds with non-toxic proteins synthetized by the producing *Clostridium*. Two classes of complex have been described based on their composition. The Ha-BoNT complexes contain some hemagglutinins (Ha). The Ha-BoNT complexes are associated to BoNT/A (subtypes A1, A5 to 8), BoNT/B, C, D, C/D, D/C, and G. Then, the Orf-BoNT complexes contain the OrfX proteins (OrfX1 to 3) and P47 protein. The Orf-BoNT complexes are associated with BoNT/A (subtypes A2 to 4), E, F and X. All complexes contain the non-toxic non-hemagglutinin protein (NTNH).

All BoNT types have the same mode of action (Popoff, 2018). This includes four steps: (i) cell binding, (ii) internalization, (iii) membrane translocation of the light chain into the cytosol after reduction of disulfide bond, (iv) enzymatic cleavage of their target soluble N-ethylmaleimide-sensitive fusion protein attachment protein receptor (SNARE).

Each BoNT enters into demyelinated terminal nerve endings by binding to presynaptic membrane receptors constituted of a membrane glycoprotein and a ganglioside (Zhang et al., 2017a). The BoNT, bound to its receptor, is internalized by receptor-mediated endocytosis inside the synaptic vesicles (Binz and Rummel, 2009). Under the effect of acid pH in the vesicles, BoNT conformation changes with the reduction of the disulfide bond and BoNT translocates its light chain into the cytosol.

The LC, a Zn^2+^-dependent endopeptidase, cleaves the SNARE proteins with extremely high specificity. The BoNT/A and E cleave the 25 kD-Synaptosomal-Associated Protein (SNAP-25). The BoNT/B, D, F, G, H and X cleave the Vesicle Associated Membrane Protein or synaptobrevin (VAMP). The BoNT/C is able to cleave the SNAP-25 and the syntaxine (Gardner and Barbieri, 2018). Table 1 reports the sites of cleavage.

**Table 1:** BoNTs targets at nervous endings and their different sites of cleavage according to toxin types.

BoNT type	Target	Peptide bond cleaved
A	SNAP25	Q^197^R^198^
B	VAMP 1–3	Q^76^F^77^
C and C/D	SNAP25	R^198^A^199^
	Syntaxin-1A	K^253^A^254^
	Syntaxin-1B	K^252^A^253^
D and D/C	VAMP	K^49^I^50^
E	SNAP25	R^180^I^181^
F	VAMP 1–3	Q^48^K^49^
	VAMP 2	Subtype F5: L^54^E^55^
G	VAMP 1–3	A^82^A^83^
H	VAMP 1–3	L^54^E^55^
X	VAMP 2–5	R^66^A^67^

The cleavage of the SNARE inhibits the release of neurotransmitter acetylcholine. Then, the inhibition of the neurotransmission leads to a block of the neuronal activation of muscles resulting in flaccid paralysis. However, depending on the cleaved SNARE (VAMP, SNAP25 or syntaxin), the inhibition of neurotransmission varies in intensity and duration. Indeed, the BoNT duration of action is different depending on the type of toxin, ranging from weeks to several months. Table 2 reports the duration of action for the different types.

**Table 2:** Duration of the effect of different types of BoNT in mice (Pellett et al., 2015, 2018a,b; Gardner and Barbieri, 2018).

Type	Duration
A	<9 months
B	2–4 months
C	4–6 months
D	3 weeks
E	2–3 weeks
F, G, H, and X	Not determined

BoNT, Botulism neurotoxin.

The BoNTs are the most potent toxins known. They are effective on human and animals, mainly mammalians and birds. Rossetto and Montecucco (2019) summarized the lethal dose values in mice by intraperitoneal route. Table 3 reports these lethal doses in mice. In mice, the toxicity is very similar by intravenous, intraperitoneal and intramuscular injection. By oral route, the lethal dose is at least a thousand times greater.

**Table 3:** Lethal dose 50 of the different types of BoNT in mice.

BoNT type	i.p. LD50 (ng.kg^−1^)
A1	0.25–0.45
A2	0.11–0.53
A3	0.85
A4	400–500
A5	0.35–0.4
A6	0.26–0.3
B1	0.21–0.5
B2	0.4
C	0.92–2.3
C/D	0.8–1.92
D	0.02–0.83
D/C	0.05
E1	0.65–0.84
E3	3.05
F1	2,4–3.6
G	5
H	1.3–2.2

i.p., intra peritoneal; LD, Lethal Dose; BoNT, Botulism neurotoxin.

### 1.3. Detection methods

Clinical signs of botulism are evocative but not specific and laboratory analysis is required to firmly confirm the suspicion (Anniballi et al., 2013b). Two main strategies are currently applied to confirm botulism. One is based on the detection of BoNT in samples while the other one is based on the detection of BoNT-producing clostridia in samples. Such analyses, during epidemiological investigations, after an outbreak in animals for example, may also contribute to identify the source of contamination or the dissemination routes. This section presents methods mostly implemented in the detection of BoNT and BoNT-producing clostridia.

Each method has advantages and disadvantages. A combination of several methods is a good way to consolidate a diagnostic. However, this approach is costlier and may delay final results. This may be prohibitive under field conditions, especially for animal botulism.

The mouse bioassay (MBA) is still considered the gold standard in BoNT detection. Since BoNT is responsible for the clinical signs, BoNT detection is still considered the best way to confirm the diagnosis. MBA is indeed one of the most sensitive test available (10 pg/ml). MBA allows for the detection of BoNT activity and not only its presence. Nevertheless, MBA has major drawbacks, such as ethical issues and a few-day delay to get results. Moreover, there is no international standardization regarding sample preparation, interpretation of clinical signs, the number of mice to be tested, their genetic background, as well as age and weight (Lindström et al., 2010). Alternative methods, such as ELISA tests (Hansbauer et al., 2016; Masters and Palmer, 2021) and Endopep-Mass Spectrometry (MS) (Björnstad et al., 2014; Drigo et al., 2020; Frye et al., 2020), have been developed and these have a lower limit of detection than MBA. However, the modalities of the validation of ELISA tests targeting either BoNT or antibodies against BoNT in animal blood are not always provided in published studies or does not always follow an official standardized protocol (Worbs et al., 2015). Moreover, controls are not systematically present, and this raises questions about conclusions that can be drawn from such tests (Lindström et al., 2006; Worbs et al., 2015). Attention should also be paid about the type of antibodies used in the ELISA tests (Lindström et al., 2006; Worbs et al., 2015). While ELISA tests will detect the presence of BoNT, Endopep-MS, like MBA,detects BoNT enzymatic activity, using synthetic peptides and further identification of cleavage product by either immunologic tests or mass spectrometry (Barr et al., 2005).

BoNT-producing clostridia are mostly detected using PCR assay after an enrichment step by culture (Anniballi et al., 2013a; Le Maréchal et al., 2017). Several methods have been published in the literature (Lindström et al., 2010). However, there is no universally agreed upon protocol for the detection of BoNT-producing clostridia in animals (Lindström et al., 2010). While a standard for a PCR detection of BoNT-producing clostridia (type A, B, E, and F) in food does exist (ISO/TC 34/SC 9 Microbiology, 2013), no reference text is available for the detection in animal samples or, more broadly, for PCR detection methods for clostridia producing BoNT/C, D, C/D, and D/C (Lindström et al., 2010). The enrichment step, preceding DNA extraction and detection, which impact the number of bacterial cells available would also deserve standardization. Moreover, as underlined by Lindström et al. (2010) and to our knowledge at the time of publication, there is no selective medium available to isolate *C. botulinum* group III (Lindström et al., 2010).

Recently, reverse-transcription real-time PCR assays targeting BoNT/C and D genes have been developed. It consists in the detection of BoNT complex–associated toxin gene RNA/DNA as an indirect method for the detection of BoNT presence in a sample (Masters and Palmer, 2021).

France relies mainly on two methods, MBA and PCR, implemented in two reference laboratories (the National Reference Center that is involved in human and animal botulism diagnosis and the National Reference Laboratory that is involved in animal botulism) (Vanhomwegen et al., 2013; Le Maréchal et al., 2017). In humans, botulism is usually confirmed by BoNT detection in serum, gastric samples, feces, vomitus or suspected food and/or by the detection of BoNT producing clostridia in feces or suspected food (Maupas et al., 1976). Attention should be paid to collect specimen(s) immediately for biological diagnosis confirmation as soon as botulism is clinically suspected. In particular, serum should be collected when BoNT is circulating, i.e., at the earliest at the onset of clinical signs and before the administration of antitoxin treatment (Curtiaud et al., 2022). In animals, the sampling material will vary depending on species. When analyzing serum, BoNT detection are often negative in bovines (Bano, 2019), turkeys (Le Maréchal et al., 2016a) as well as in horses (Johnson et al., 2015), even in animals with clinical signs. Analysis of other samples such as fecal material and organ tissue, using PCR assay or by use of a combination of methods to detect both BoNT and BoNT-producing clostridia markedly increases the reliability of the diagnosis. In bovine, the liver, ruminal or intestinal content, manure, suspected feed or environmental samples can be tested. The liver is recommended for avian species (Le Maréchal et al., 2016a). In fish, the presence of BoNTs in blood or the digestive tract, and the detection of BoNT-producing clostridia in the digestive tract can be used to confirm botulism. The use of *Danio rerio* (zebra fish) instead of mice for BoNT detection in fishes has been suggested because of its higher sensitivity to BoNT/E (Chatla et al., 2014, 2016).

## 2. *Clostridium botulinum* and its environment

### 2.1. Distribution and prevalence

*Clostridium botulinum* is a spore-forming anaerobic bacterium widely distributed in nature (Long and Tauscher, 2006; Espelund and Klaveness, 2014). *C. botulinum* can be found in raw water storage areas and waste water (Anza et al., 2014), trout farms (Cann and Taylor, 1982; Eklund et al., 1984), fish and environmental samples from coastal and wetland areas. Forage botulism or feed-related botulism seems to be an important pathway for farm animals. The species is present in all continents except Antarctica to our knowledge, in soil, dust, water and marine and freshwater sediments. The spores can persist in soils and sediments for decades (Long and Tauscher, 2006). *C. botulinum* is occasionally present in the intestinal content of healthy animals (fish, birds and mammals) (Espelund and Klaveness, 2014). The worldwide distribution and prevalence of *C. botulinum* differs according to regions for their toxin type. However, the ecological factors that determine the distribution remain unknown. For group I, *C. botulinum* type A occurs at a higher frequency in prevalence studies performed in the western United States while *C. botulinum* proteolytic type B dominates in the eastern United States (Shapiro et al., 1998). Regarding group II, non-proteolytic *C. botulinum* predominates in western Europe. *C. botulinum* type C and D are commonly found in Europe and in Australia (Woudstra et al., 2012; Masters and Palmer, 2021). *C. botulinum* E is predominately identified in aquatic habitats in northern Europe, in the United States and Canada, noteworthy in the Great Lakes region, and in the North Pacific area (Bott et al., 1966, 1968; Laycock and Loring, 1972; Horowitz, 2010; Austin and Leclair, 2011; Leclair et al., 2013, 2017). In Asia, types A to F are widely found in China (Yamakawa et al., 1988; Gao et al., 1990; Fu and Wang, 2008) but the presence of only types B, C and E was described in Japan (Yamakawa et al., 1988; Yamakawa and Nakamura, 1992; Umeda et al., 2013). All types except E are found in Indonesia (Suhadi et al., 1981). Types A, B and D are present in Australia (Eales and Turner, 1952; Murrell and Stewart, 1983; Koepke et al., 2008). In Africa, types A to D are detected in Zambia and Kenya (Nightingale and Ayim, 1980; Yamakawa et al., 1990; Karasawa et al., 2000).

### 2.2. Ecology

Certain plants (in particular algae) and invertebrates (in particular naturally resistant mollusks and insect larvae) can store types C, D, C/D, or D/C and type E toxins in their tissues or cells, and they have been identified as mechanical vectors (Hubálek and Halouzka, 1991; Rocke et al., 1999; Anza et al., 2016). Moreover, fish and birds can harbor the bacteria in their digestive tract as observed in the USA (Bott et al., 1968), and, depending on their sensitivity, may develop the disease. The decomposition of plant species and contaminated animal carcasses (such as fish, birds and mammals) is an optimal condition for bacterial development and toxin production. These conditions facilitate the transmission of the bacterium to species that are sensitive to the toxin, such as livestock (poultry, cattle) and humans consuming contaminated products.

Types C, D and mosaics C/D or D/C are, in nature, closely associated with wetlands rich in sediments (e.g., marshes, ponds and lakes) which are favorable to the bacterial development (Wobeser et al., 1987). The contamination of susceptible birds and the initiation of a “carcass-maggot” amplifying cycle can lead to the development of outbreaks of botulism in the avifauna (Wobeser, 1997; Soos and Wobeser, 2006). Type E, mainly identified in the northernmost regions of the northern hemisphere, is closely associated with aquatic, marine or freshwater ecosystems (Dolman, 1957; Haagsma, 1991; Horowitz, 2010; Espelund and Klaveness, 2014; Palmer et al., 2019). Carriage by fish is most often intestinal (Bott et al., 1966, 1968; Yule et al., 2006); the contamination can lead to mortality in certain species more sensitive to toxin E. The disease can also spread to aquatic or coastal birds (fish-eating birds in particular), sometimes causing the death of thousands of individuals (EPA, 2013).

*Clostridium botulinum* eradication is inconceivable because of its close association with natural environments in which it survives or evolves. It appears that the lethal outbreaks of botulism occasionally affecting the avifauna exposed to types C, D, C/D, or D/C and E result from multiple factors (weather, organic pollution generated by human activities, etc.) that contribute to the disruption of ecosystems and make them more favorable to outbreak occurrence (Sandler et al., 1993; Rocke et al., 1999; Lafrancois et al., 2011). Knowing these factors can make possible the identification and the implementation of control management measures (in particular the collection and destruction of animal carcasses in areas at risk) (Inger et al., 2016; Reynolds et al., 2021). Some studies demonstrated an interaction of environmental conditions (temperature, pH, sodium chloride and organic matter concentrations) affecting spore germination, outgrowth and toxin formation and proposed applied mathematical models (Barras and Kadlec, 2000; Chea et al., 2000; Zhao et al., 2003).

The prevalence of C, D, C/D, or D/C and E toxin types in agricultural fields seems quite low, considering the rare data regarding the digestive carriage in livestock and the presence of spores in farm environments (grassland, cultivated land) far from wetlands for types C, D, C/D, or D/C, or far from contaminated coasts, lakes and rivers for type E (Smith and Milligan, 1979; Gessler and Böhnel, 2006). Although relatively infrequent (even for type E), the emergence of cases of botulism in herds or flocks (often infected *via* contaminated feed or water) are worrisome.

## 3. Zoonotic aspects

### 3.1. Human botulism forms

Human botulism is a serious and potentially lethal disease. BoNT is the most potent bacterial toxin and definitely one of the most potent known poison (Arnon et al., 2001; Kumar et al., 2016). Botulism occurs worldwide but the number of reported cases varies between regions and countries. This variation may be due not only to real differences in incidence, but also to underreporting.

Several types of botulism are described in humans, depending on the mode of contamination and exposure to the toxin. Foodborne botulism, infant botulism, adult botulism, wound botulism, inhalation botulism and iatrogenic botulism are thus reported (Anniballi et al., 2013a).

Foodborne botulism (poisoning) is the main cause of human botulism in Europe (Popoff, 2018). It results from digestive intoxication due to the presence of the preformed toxin in the food. It is present on all continents and is of variable incidence. Overall, foods associated to foodborne botulism are very diverse (Lund and Peck, 2013). In Europe, it mainly involves homemade preserved products. Food of concern are mainly cured meats and canned vegetables, in Europe (Meurens et al., 2021; Le Bouquin et al., 2022). In a few rare cases, commercial foods or restaurant meals have been involved. After ingestion, the toxin is absorbed by the duodenum and jejunum and then passes into the bloodstream. BoNT resists gastric acidity and digestive enzymes because it forms a complex with a group of neurotoxin associated proteins or NAPs (Chellappan et al., 2014). BoNT/A, B and E are associated with foodborne botulism.

Infant botulism (toxi-infection) occurs when spores of *C. botulinum* are ingested from the environment or with honey (Popoff, 2018). Then, the bacteria germinate and multiply in the gastrointestinal tract and release the toxins (A, B, or F) produced *in situ*. This form of botulism has been reported in children from 6 days to 12 months of age, but mostly in infants from 2 to 8 months of age. Small doses of spores (10–100) are sufficient to induce intestinal colonization and toxin production (Popoff, 2018). The intestinal microbiota, which has normally an inhibitory effect on the growth of *C. botulinum*, does not play its inhibitory role in infants below the age of one (Popoff, 2018; Douillard et al., 2022).

Adult botulism (toxi-infection) is a rare and poorly understood form of botulism similar to infant botulism but occurring in adults (Fenicia et al., 2007; Freund et al., 2017; Harris et al., 2020). It usually involves type A toxins, but type B and F toxins have sometimes been implicated. In these patients, spores, bacteria, and toxins are found in the stool and spores may also be found in leftover food, but no preformed toxins are found. Dysbiosis is suggested but the exact causes of this alteration of the intestinal microbiota remain unknown. The intestinal microbiota, normally well established and fully functional after infancy, prevents bacterial colonization of the digestive tract. Possible imbalance of the microbiota (immune depression, prolonged antibiotic use or intestinal surgery) could be involved.

Wound botulism (inoculation) is a consequence of the contamination of wounds by *C. botulinum* spores (Popoff, 2018). At the vicinity of wounds, the bacteria can grow and produce neurotoxin (mostly type A or B). Since the 1980s, this rare form of botulism has been almost exclusively linked to the use of injectable drugs (Neeki et al., 2021). Previously, cases of wound botulism occurred in open fractures, deep traumatic wounds, or puncture wounds contaminated with foreign elements. The lesions have to be deep enough to enable anaerobic conditions required for spore germination and toxin production.

Inhalation botulism is also very rare (Popoff, 2018). A few cases have been reported amongst laboratory workers preparing concentrated BoNTs by continuous centrifugation (Holzer, 1962) and in a few individuals following the intranasal use of contaminated cocaine (MacDonald et al., 1985). The presence of *C. botulinum* sinusitis or direct absorption through a nasal mucosa have been suggested as etiological hypotheses. Iatrogenic botulism (very rare) will not be presented here.

The treatment of botulism is symptomatic and involves supportive care, intubation and mechanical ventilation when needed as well as administration of botulinum antitoxin (Rasetti-Escargueil and Popoff, 2019; Rao et al., 2021). Prevention is also possible using vaccines and several approaches, including DNA-based, viral vector-based, and recombinant protein-based vaccines, have been developed and tested (Sundeen and Barbieri, 2017; Rasetti-Escargueil and Popoff, 2019).

### 3.2. Surveillance data

In Europe, botulism is monitored through the surveillance of zoonoses and zoonotic agents and the protection of workers. In France for instance, botulism is a notifiable disease, in humans, and to some extent in animals, regardless of the species affected (Le Bouquin et al., 2022). Reporting the disease has been mandatory in France for human disease since 1986 and between 2006 and 2022 for poultry, cattle, and wild birds.

If reporting of severe forms of human botulism is probably exhaustive, many animal botulism cases remain suspicious and are not formally reported, especially for wild birds. Surveillance of botulism in wild birds is based on event-based surveillance and unquantifiable.

A study conducted on human botulism surveillance in France over the last decade shows that the incidence of human botulism has been relatively stable over time with an average of ten outbreaks/year for a total of 100 cases (Le Bouquin et al., 2022). The incidence in Europe was around 0.02 cases/100,000 persons, similar to what has been observed in France. Similarly, animal botulism also appears to be relatively stable, although annual variations are observed (Le Bouquin et al., 2022). Each year, an average of 30 outbreaks are recorded in France on poultry farms, about 20 cases in wild birds and about 10 outbreaks in cattle, often involving a large number of animals. Few other animal species, including domestic carnivores, are affected by botulism in France. Botulism has been confirmed in 264 avian species representing 39 families (Rocke, 2006), among which *Anatidae* appears to be the most affected family in wild birds, at least in France (Ventujol et al., 2017). Regarding poultry, outbreaks have been reported in chicken broilers, turkeys, pheasants and to a lesser extent ducks, guinea fowls, laying hens, gooses, and quails (Souillard et al., 2014; Le Maréchal et al., 2016a, 2017; Ventujol et al., 2017).

Analysis of the toxin types occurring in France confirmed the predominance of types A and B in human botulism in both foodborne and infantile cases, and exceptionally type F (Mazuet et al., 2017; Rasetti-Escargueil et al., 2020; Le Bouquin et al., 2022). The C/D mosaic form is the predominant BoNT in birds in this country, even if BoNT/D is also observed, and only BoNT/D/C and C have been identified in recent years in cattle.

### 3.3. Lethal doses of BoNTs in humans

The preformed toxin (types A, B, E and F) are active in humans (Rasetti-Escargueil et al., 2020) after ingestion, injection, and inhalation; the skin offers protection against the toxin. The lethal doses in humans have been estimated by extrapolation from studies carried out in primates (Herrero et al., 1967; Franz et al., 1993). Table 4 summarizes these doses for the BoNT/A1 (Arnon et al., 2001). Regarding BoNT/B, Rasetti-Escargueil and collaborators (2020) indicate that the minimal toxic dose by ingestion in humans is about 4,000 mouse lethal doses (MLD) and a minimal dose of 30–100 ng of BoNT/B has also been reported to induce foodborne botulism in humans (Peck, 2009). The therapeutic use of BoNT in human has shown that the effect of BoNT/A and BoNT/B are comparable with a dose ratio 1:25 to 100 following the application (Bentivoglio et al., 2015). The other types of BoNTs are usually considered less toxic than type A but there are no estimated lethal doses available for humans. Compared to humans, the data available in monkeys by oral route (Rasetti-Escargueil et al., 2019) indicate that BoNT/B (160 MLD.kg^−1^) is the most toxic followed by the BoNT/A (650 MLD.kg^−1^), the BoNT/E (1,500 to 2,500 MLD.kg^−1^) and the BoNT/F (50,000 to 75,000 MLD.kg^−1^). The BoNT/C and D appear to be less potent for monkeys with death occurring at 100,000 MLD.kg^−1^ for BoNT/C and 600,000 MLD.kg^−1^ for BoNT/D. Otherwise, within each type, the toxicity of subtypes are different. For the BoNT/A, the subtypes A1 and A2 are the most potent and BoNT/A4 is the less toxic about a 1,000 fold compared to other subtypes A (Pellett et al., 2015).

**Table 4:** Estimated lethal doses for BoNT/A1 in humans.

Route of contamination	Estimated lethal doses (ng/kg^−1^)
Oral	1,000
Inhalation	10–15
Intravenously and intramuscularly	1–2

BoNT, Botulism neurotoxin.

### 3.4. BoNT/C, D, C/D, and D/C in humans

Outbreaks of botulism occur in cattle and poultry every year in many countries including France. These outbreaks are essentially caused by toxinotypes C, D, C/D, and D/C. Toxinotypes C, D, C/D, and D/C are generally not associated with human botulism. Nevertheless, the question of the zoonotic potential of these toxinotypes has emerged in humans. To the best of our knowledge, only an extremely limited number of cases of human botulism has been linked to toxinotypes C, D, C/D, and D/C (see Table 5). Most often, the few available data are rather old (before 2000), and more often published as conference proceedings than peer-reviewed publications in international journals. Moreover, no lethal dose for toxinotypes C, D, C/D, and DC was identified so far in humans. Some cases where botulism type C or D was suspected are presented in Table 5.

**Table 5:** Zoonotic evidences of toxinotypes C, D, C/D, and D/C mosaics.

Country Year	Cases* (deaths)	Biological samples analyzed*: Results	Suspected or confirmed source** Food samples analyzed: Results	Human botulism Outbreak
USA, 1950	4 (1)	Stomach fluid: Presence of BoNT/C and *C. botulinum* type C	None**	Clinical suspicion type C
France, 1955	2 (0)	None*	“*Pâté*” (Homemade): Absence of BoNT Presence of *C. botulinum* type C	Clinical suspicion type C
Chad, 1958	2 (0)	None*	Ham (Homemade): Presence of BoNT/D and *C. botulinum* type D	Clinical suspicion type D
Rhodesia, 1960	4 (0)	None*	“*Pâté*” (Homemade): Absence of BoNT Presence of *C. botulinum* type B or C	Clinical suspicion type B or C
**France**, **1972**	**4 (1)**	**Sera: Presence of BoNT/C**	**Smoked chicken suspected (not analyzed)**	**Foodborne botulism type C**
**Japan**, **1990**	**1 (0)**	**Stool: Presence of BoNT/C**	**Environmental contamination suspected (no samples analyzed)**	**Infant Botulism type C**
France, 2006	1 (0)	Serum: absence of BoNT Stool: Absence of BoNT and *C. botulinum*	Consumption of sick chicken before onset of symptoms	Clinical suspicion type C or D

BoNT, Botulinum neurotoxin. In bold in the table, confirmed case of botulism. *Case: Having shown at least one typical sign of botulism: diplopia, mydriasis, swallowing difficulties, dry mouth, slurred speech, paralysis, constipation (vomiting and diarrhea are not specific to botulism). **Suspected or confirmed sources.

Maupas et al. (1976) detected BoNT/C in two out of four sera from patients suffering from botulism but excluded the hypothesis of intestinal botulism, explaining that *C. botulinum* type C could not produce toxins at 37°C. Thus, the hypothesis of foodborne botulism involving smoked chicken was preferred even if smoked chicken samples were never analyzed. In 1990, infant botulism type C was confirmed by the detection of a huge amount of BoNT/C in a stool sample collected from the patient suffering of botulism (Oguma et al., 1990). However, again and as for almost all cases of infant botulism, the origin could not be determined. In a recent case, the symptoms of botulism were concomitant with an outbreak of type C botulism in a family poultry farm, without biological confirmation in the patient (Martrenchar et al., 2019).

In parallel, type C botulism has been confirmed in primates (Dolman and Murakami, 1961; Smart et al., 1980; Silva et al., 2018) in facilities linked to the preparation of meals based on poorly preserved or poorly thawed poultry.

Type D botulism following the consumption of ham was suspected in Chad and presented in a communication to the French Academy of Medicine (Demarchi et al., 1958). BoNT and *C. botulinum* type D were detected in samples from this ham. However, the absence of further investigation in the patients and the conditions of sample collection (heterogeneity, delays, climatic conditions, transport, culture, etc.) impaired the confirmation of the zoonotic character of this outbreak.

Several hypotheses have been put forward to explain the near absence of human cases of types C, D, C/D, and D/C botulism: low host susceptibility, low human exposure or lack of surveillance. Low human susceptibility to C, D and mosaic toxins is the preferred hypothesis (Meurens et al., 2021). However, *in vivo* tests carried out using intramuscular route showed the “efficacy” of the toxin (particularly type C) (Eleopra et al., 1997, 2013). Thus, the low sensitivity could correspond to a low intestinal absorption of the toxins.

## 4. Botulism and animals

### 4.1. Epidemiological cycles

*Clostridium botulinum* is a bacterium present in both the digestive tract of animals and in the environment (soil, water, sediment, etc.) (Long and Tauscher, 2006; Le Maréchal et al., 2016b). Its ubiquitous character enables different possibilities to enter cattle herds and poultry flocks.

#### 4.1.1. Epidemiological cycle of *Clostridium botulinum* in cattle farms

**Figure 2:**
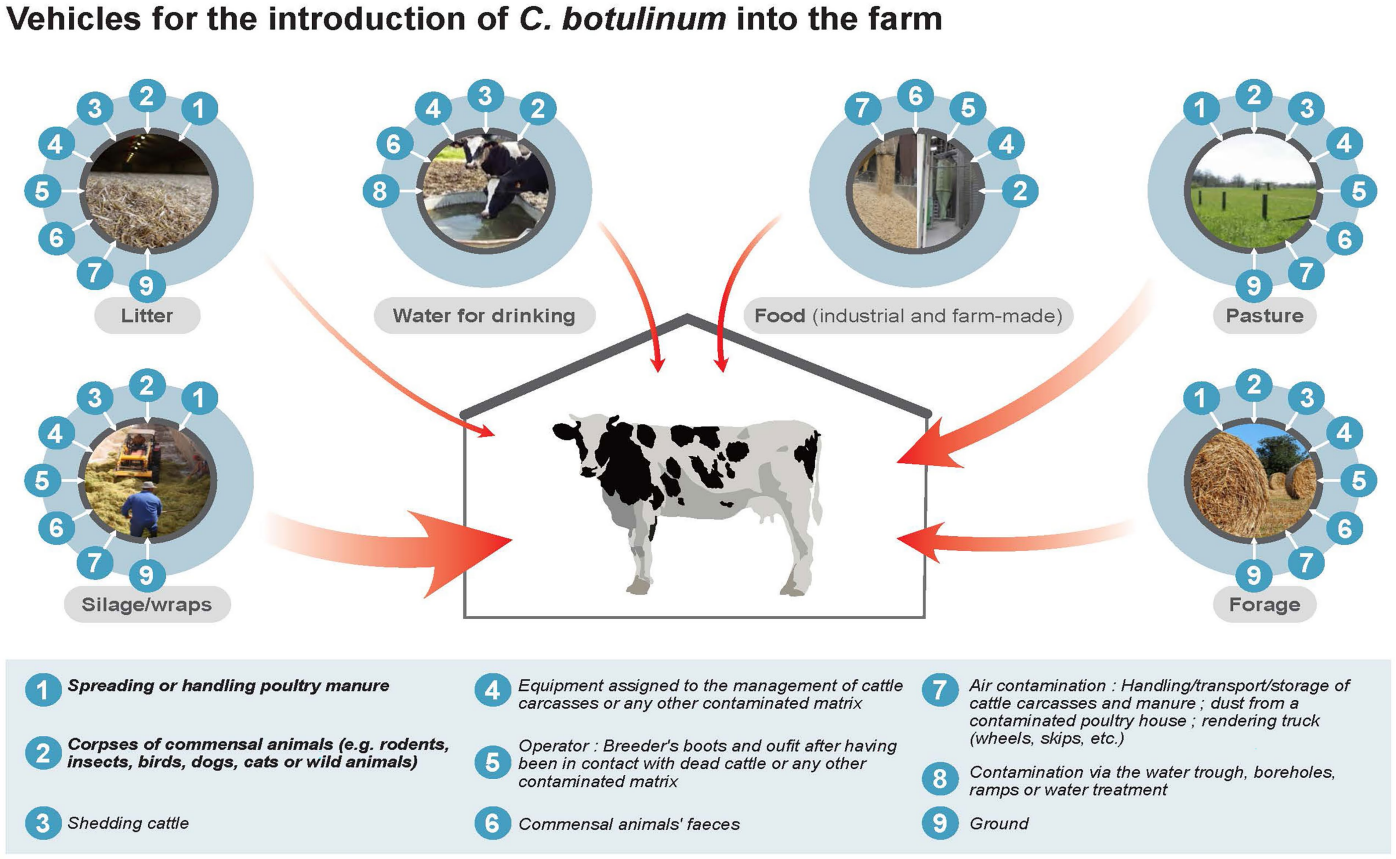
Epidemiological cycle of *Clostridium botulinum* in cattle farms.

Clinical signs observed in cattle are due to ingestion of BoNTs and/or BoNT-producing clostridia by animals *via* drinking water (Doutre, 1969) or feeding (pastures (Popoff, 1989), forages (Bano et al., 2015) and on-farm manufactured feeds (Le Maréchal et al., 2019), silage (Myllykoski et al., 2009; Guizelini et al., 2019), or haylage previously contaminated with *C. botulinum* (Relun et al., 2017). Two major sources of contamination leading to bovine botulism outbreaks have been identified. One is the presence of animal carcasses (domestic or wild) that provide a substrate for the development of *C. botulinum* and production of BoNT (Le Maréchal et al., 2019). The second one is poultry manure, as poultry can be asymptomatic carriers of *C. botulinum* (Souillard et al., 2017, 2021). Contamination of feed or water by these two sources can occur directly with the presence of the dead animals or manure in the drinking water or in the feed. Contamination can also occur indirectly *via* contaminated equipment (e.g., tractor bucket), clothing [farmer, technician, veterinarian (Souillard et al., 2017, 2021)], or by airborne route (Hogg et al., 2008). Soil and feces, through intestinal carriage by animals, not only livestock but also other domestic or wild animals, can also be a source of contamination (Figure 2).

Table 6 presents the prioritization of different initial sources and contamination vehicles of *C. botulinum* (spore and vegetative forms and toxins) in a cattle farm, in decreasing order of importance. Contaminated poultry manure appears therefore to be the main vehicle of contamination of *C. botulinum*, with a direct or indirect link between the cattle farm and a poultry flock. The source of manure contamination is asymptomatic poultry carrying *C. botulinum*, and excreting it into the litter (Souillard et al., 2017, 2021). Contamination of water or feed by a dead animal is also a major source of contamination for outbreaks of bovine botulism (Galey et al., 2000; Le Maréchal et al., 2019). Regarding commercial feed, drinking water and cattle litter, these vehicles of contamination appear to be less important, due to good management practices that favor their control (discarding of manure and carcasses, storage conditions of feed, cleaning of equipment, no reuse of poultry litter...).

**Table 6:** Prioritization of importance (decreasing order) of the different initial sources and vehicles of *C. botulinum* contamination (spore, vegetative form and toxins) in a cattle farm (Anses, 2021).

Initial source of contamination	Source of contamination	Argumentation for prioritization
Asymptomatic (or symptomatic) carriage by poultry and excretion in effluents	Poultry manure	Quantitative importance of this source of contamination in France with (direct or indirect) link between the cattle farm and a nearby poultry farm
Carcasses of dead animals	Contaminated silage or wrapping	Inclusion of an animal during harvest
Equipment		Dual role of equipment: - Contamination of silage if not cleaned. - Homogeneous distribution of the contamination throughout the ration (e.g., with a silo mixer)
Animal feces, operators, airborne contamination		Lesser importance than dead animals and equipment
Spreading of poultry manure	Pasture	Contamination of cattle if contaminated poultry manure is spread on pasture (direct ingestion) or if a dead animal is present
Carcasses of dead animals		
Carcasses of dead animals	Other forages/ On-farm feed fabrication	Contamination of the feed if a dead animal or contaminated raw material is present
Contaminated raw materials		

#### 4.1.2. Epidemiological cycle of *Clostridium botulinum* in poultry flocks

**Figure 3:**
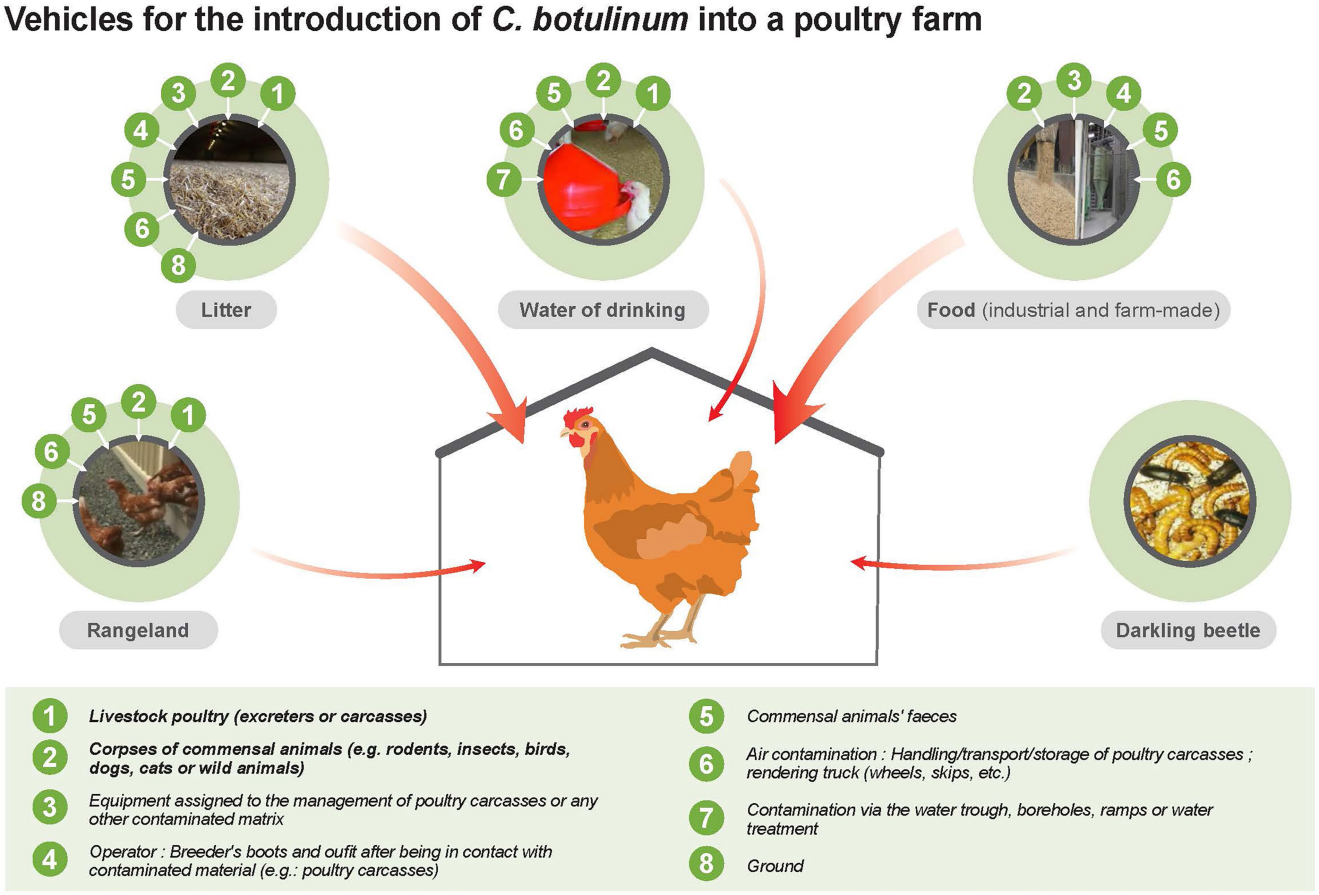
Epidemiological cycle of *Clostridium botulinum* in poultry flocks.

In poultry flocks, botulism outbreaks are mainly associated with carriage of *C. botulinum* by poultry (Dohms, 2008) and with any other animal species (rodents, wild birds and others) outside the farm that may enter and contaminate litter and/or feed *via* feces or carcasses (J.P. Vaillancourt, personal communication) (Figure 3; Table 7). Clothes and boots of personnel (e.g., farm employees, catchers, technicians) and the equipment used in the barns were also reported as possible sources of contamination (Figure 3).

**Table 7:** Prioritization of importance (decreasing order) of the different initial sources and vehicles of *C. botulinum* contamination (spore, vegetative form and toxins) in a poultry flock.

Initial source of contamination	Source of contamination	Argumentation for prioritization
Carcasses of dead animals	Feeds	Inclusion of an animal during harvest or storage of feed in silo
Equipment		
Feces of commensal animals		Lesser importance than dead animals and equipment
Excreting animals in the flocks	Litter	- Litter contamination with feces - Asymptomatic carriage of *C. botulinum* in poultry (unknown prevalence)
Contaminated equipment		If equipment used to handle the litter is contaminated, there is a risk of contamination and introduction into the barn
Carcasses of dead animals and/or poultry		Inclusion of an animal carcass during harvest
Carcasses of dead animals and/or poultry	Drinking water	Contamination of drinking water with a dead animal carcass
Equipment		- Equipment, if contaminated or not cleaned, can contaminate drinking water. - Point-source contamination is widely spread in drinking water by contaminated equipment
Animal feces		Lesser importance than dead animals and equipment

Table 7 presents the different initial sources and vehicles of contamination of *C. botulinum* (spore, vegetative form and toxins) in a poultry flock and their prioritization. The feed appears to be the main vehicle of contamination of *C. botulinum*. Feed can be contaminated after inclusion of an infected animal at harvest or during storage in silos. In case of on-farm feed production, the harvest equipment can also be a vehicle of contamination (e.g., the same trailer for manure hauling and harvesting). For commercial feeds, good management practices are very important for controlling these fomites (Anniballi et al., 2013a). Litter (e.g., rice hulls) also appears to be a contamination vehicle for *C. botulinum* in poultry flocks (Bano et al., 2013; Fillo et al., 2021). Litter should be protected from wild bird droppings. Wild birds can be asymptomatic carriers of *C. botulinum,* although the prevalence and conditions of this carriage is unknown (Reed and Rocke, 1992; Reynolds et al., 2021).

### 4.2. Bovine botulism

Bovine botulism is mostly due to BoNT/C, D, C/D, and D/C (Woudstra et al., 2012; Bano, 2019) although some type A (Schocken-Iturrino et al., 1990; AFSSA, 2002; Rasetti-Escargueil et al., 2019; Frye et al., 2020) and B (Notermans et al., 1981; Divers et al., 1986; Wilson et al., 1995; Kelch et al., 2000; Yeruham et al., 2003) cases are sporadically reported in the literature. Presently, BoNT/D/C is the most common BoNT type involved in bovine botulism outbreaks in Europe.

Bovine botulism is considered to be due to the ingestion of pre-formed BoNT present in feed, water or any other sources of toxin. Animal carcasses, poultry manure as well as feed inappropriately stored provide favorable conditions for the growth of *C. botulinum* and the production of BoNTs. The role of *C. botulinum* cells themselves in the pathogenesis of bovine botulism needs to be clarified. Currently, the PCR is commonly used for the laboratory confirmation of bovine botulism through detection of the bacteria in enrichments of the liver, rumen, intestinal contents or other sample collected on symptomatic or dead animals. A biphasic distribution of mortalities may be observed. Acute clinical signs are noted in the herd 3–4 days after exposure and another set of clinical signs are recorded about 14–20 days after the first clinical observations. This clinical pattern may be linked to *in situ* BoNT production: a first wave of deaths being due to the ingestion of BoNT and a second wave being linked to *in situ* BoNT-production. This hypothesis is supported by the detection of vegetative cells in livers collected on animals that died from botulism (Le Maréchal et al., 2019).

Bovine botulism is characterized by a flaccid paralysis, generally starting by the paralysis of the tail and hind legs, then progressing to the head (Kummel et al., 2012; Bano, 2019). The incubation period varies between a couple of hours and 2 weeks. Three forms can be encountered: a peracute form with sudden lateral recumbency and death within a few hours after exposure; an acute form with typical clinical signs (anorexia, ataxia, apathy, weakness, dysphagia, increased salivation, paralysis, recumbency with head tucked into the flank, and dropping feed from the mouth) and death within 2–3 days after the onset of the disease; and a subacute form with attenuated clinical signs, from which animals might sometimes recover (mainly observed for type C outbreaks). Usually, major economic losses are associated with bovine botulism in cattle farms (up to 100% mortality rate). The differential diagnosis must include hypocalcemia, hypophosphatemia, some types of enterotoxemia, listeriosis, paralytic rabies, organophosphate or lead poisoning (Dorchies and Leze, 2017). Besides clinical signs, the diagnosis is based on the epidemiological context, in particular the suspicion of high risk sources such as poultry manure or the detection of a dead animal in the feed. As for other species, the diagnosis should be confirmed by the detection of BoNT and/or BoNT-producing clostridia in samples collected on animals or their closed environment. The analysis of several samples collected on various animals can be required in order to detect BoNT and/or BoNT-producing clostridia to confirm the diagnosis.

There is currently no curative treatment available for bovine botulism, although antitoxins might be successfully used, but at costs usually not sustainable for most farms (Guizelini et al., 2019). Vaccination can be used using toxoids targeting BoNTs. Several vaccines have been developed worldwide (Anniballi et al., 2013b) but market authorizations have been issued by only a few countries and may be only temporary as in France with a sole authorized vaccine. This “French” vaccine is only used for emergency vaccination to protect healthy animals in farms facing a botulism outbreak or to prevent disease recurrence. To prevent bovine botulism outbreaks, it is crucial to implement biosecurity measures as far as farm management and feed production and storage are concerned. The presence of animal carcasses in the feed (silage, haylage, cereals…) or feed stored under bad conditions (uncovered, exposed to wild bird droppings for example) may be the source of an outbreak. It is crucial to pay attention to all steps from feed harvesting to animal distribution. The implementation of dedicated biosecurity measures between poultry and cattle is essential to prevent cross-contamination between the two productions. The equipment needs to be cleaned and disinfected; in mixed farms, clothes and shoes should be changed between both productions.

### 4.3. Avian botulism

Botulism is widespread in most avian species, both wild and domestic. On a worldwide basis, avian botulism is the most significant disease of water birds. Some species, especially scavengers like vultures, are known to be resistant. For still unknown reasons, males tend to be more affected than females, in particular in turkey farms (Smart et al., 1983; Popp et al., 2012; Souillard et al., 2014).

Avian species are sensitive under experimental conditions to all BoNT types by intravenous route, with differences being observed among avian species and depending on doses (Gross and Smith, 1971; Miyazaki and Sakaguchi, 1978). In wildlife and in farm environments, only BoNT/C, D, C/D, D/C, and E have been involved in avian botulism outbreaks, and BoNT/A to a much lesser extent (Rogers et al., 2021). The most common BoNT type at the moment, at least in Europe, is C/D (Woudstra et al., 2012; Souillard et al., 2014; Le Maréchal et al., 2016a).

Whether avian botulism is due to the ingestion of preformed BoNTs or to *in situ* BoNT production by *C. botulinum* is not completely clear and it has been suggested that both sources of BoNT may likely coexist during an outbreak (Skarin et al., 2015). In broilers, it is assumed that BoNT is produced *in situ* in the caeca, as a large amount of toxins is necessary to get clinical signs in this avian species. The amount of BoNT normally present in the immediate environment of a flock is thought not to be high enough to cause botulism (Dohms et al., 1982; Popoff, 1989). Experimental studies have confirmed the importance of the caeca in the expression of the disease (Miyazaki and Sakaguchi, 1978; Kurazono et al., 1987; Hyun and Sakaguchi, 1989). Spores are ingested by the birds, then germinate, multiply and produce BoNT in the caeca. Spores, vegetative cells as well as BoNTs are then excreted in the litter and re-ingested by birds *via* coprophagy. BoNT enters blood circulation and reaches nerve endings, which leads to paralysis (Miyazaki and Sakaguchi, 1978; Kurazono et al., 1987; Hyun and Sakaguchi, 1989). Several studies have shown that *C. botulinum* can be detected in many organs (liver, spleen, crop) in affected birds, but the potential role of these organs colonization in the observed clinical signs or in the pathogenesis is unknown (Dohms et al., 1982; Franciosa et al., 1996; Anniballi et al., 2012, 2013a; Woudstra et al., 2012). Risk factors behind the initiation of a botulism outbreak are not clearly understood, in particular the role of asymptomatic carriage of *C. botulinum* by birds. One hypothesis is the ingestion of BoNT from bird carcasses. This hypothesis implies the presence of *C. botulinum* in the digestive tract of birds before their death. Although demonstrated, the prevalence of this carriage is highly variable. A study including 100 broiler flocks failed to detect *C. botulinum* type C/D in caeca (Hardy and Kaldhusdal, 2013), confirming another previous study where litters from 100 poultry farms tested negative for *C. botulinum* (Roberts and Aitken, 1974). In contrast, *C. botulinum* type C/D was detected in 11% of healthy poultry flocks in Sweden (Blomqvist et al., 2010) and in one farm out of 23 examined in France (Souillard et al., 2013). The detection of *C. botulinum* type D/C has been reported several times, in particular thanks to connections with a bovine botulism outbreak (Le Maréchal et al., 2020; Souillard et al., 2021). Variability in prevalence of avian botulism may be explained by the existence of *C. botulinum* carriage at a very low level, likely underestimated considering the low sensitivity in tested samples of currently available methods (Popoff, 1989; Le Bouquin et al., 2017).

Clinical signs in avian species are flaccid paralysis progressing from the legs to the nictitating membranes and often associated with respiratory failure. The first sign in broilers is leg followed by wing paralysis (Bano, 2019). One of the typical clinical signs is the paralysis of the bird’s neck, which has resulted in the use of “limber neck” to describe botulism. High mortality rates are reported. In France, the mean mortality rate was 13.9% in 17 investigated poultry botulism outbreaks, ranging from 2.8 to 35.2% in one study (Souillard et al., 2014) and between 1 and 25% in another study (Dohms et al., 1982). Higher mortality rates have been reported in the literature in case reports: 30 and 50% in turkey farms (Smart et al., 1983; Popp et al., 2012) and 84% in a pheasant farm (Borland, 1976). On a worldwide basis, avian botulism is the most significant disease of waterbirds (Rocke, 2006). Outbreaks with more than 100,000 dead birds have been reported in the USA (Rocke and Bollinger, 2007). Differential diagnosis should include ionophore intoxication (in particular in turkeys), lead as well as selenium intoxication, Marek disease and avian Flu. Diagnosis is based on clinical signs, the epidemiological context and laboratory results (detection of BoNT or/and BoNT-producing clostridia in samples collected on animals, feed or close environment).

Beta-lactams are successfully used in poultry farms during botulism outbreaks (Anniballi et al., 2013b). Several practices can be implemented to mitigate the lethal incidence of an outbreak: physical separation of symptomatic from non-symptomatic birds, replacing litter at higher frequency and fresh litter addition to prevent ingestion of BoNT and BoNT-producing clostridia, and regular disposal of birds found dead in barns. Vaccination can be considered, but does not seem to be commonly use in farms. Regarding wild birds, sick individuals can be cared for by feeding and watering them until disappearance of clinical signs.

Implementation of biosecurity measures in poultry farms is crucial to prevent both the initiation and recurrence of outbreaks: rodent control, proper feed storage conditions and feed distribution, and regular dead bird disposal. Due to the high resistance of spores in the environment, special attention should be paid to cleaning and disinfection operations (with sporicides, in both barn and equipment) after an outbreak of botulism to avoid recurrence. In wild birds, removal and proper disposal of dead birds is the most effective measure to prevent or mitigate outbreaks.

### 4.4. Control by food processing operations and during food storage

Contamination of food of animal origin, at least of raw materials, with *C. botulinum* type III is documented (Dodds, 1993a,b; Le Maréchal et al., 2016b). This contamination justifies that control measures should be implemented for safe food production. The principles of control of microbial hazards in food are determined by the data and parameters describing their sensitivity to physical or chemical inactivation treatments, their ability to multiply and, where appropriate, produce toxins in defined conditions. Compared to *C. botulinum* groups I and II, which have long been recognized as the main causes of foodborne botulism, there is very little information defining the principles of control of *C. botulinum* type III strains. For thermal treatments under moist heat conditions commonly applied in food processing, the decimal D reduction times at 104°C of spores of group III strains (*n* = 4) have been estimated to be 0.1–0.9 min (Segner and Schmidt, 1971) and about 12 min (Portinha et al., 2022) at 90°C (*n* = 2). These sparse data suggest a resistance to moist heat much higher than that of spores of non-proteolytic *C. botulinum* group II, but also a much lower resistance than that of spores of proteolytic *C. botulinum* group I. The sensitivity to temperature changes expressed by the *z*-value (temperature increase in °C resulting by 10-fold reduction in *D*-values) was estimated to be 5.0–6.2°C, i.e., close to *z* = 6.9°C of group II strains and lower than *z* = 11.3°C of group I strains established in meta-analyses (Diao et al., 2014; Wachnicka et al., 2016). This practically means that pasteurization treatments will be ineffective against *C. botulinum* type C spores as they are for other spore-forming bacteria. It also means that heat treatments to control *C. botulinum* group I (i.e., “botulinum cook” for 3 min at 121°C, or any treatment with similar lethality) will also control *C. botulinum* group III. BoNTs of types C and D seem to be more heat resistant than those of types A, B, E. However, heat treatments higher than 90°C/2 min allow total inactivation of these toxins (Roberts and Gibson, 1979).

For radiation treatments, a D10 value of 2.1 KGy was established for type C spores, not dramatically different from D10 = 3.3 kGy for group I spores and D10 = 1.4 kGy for group II spores (Desmonts et al., 2001). Unfortunately, no data are available regarding the resistance of group III spores to common food industry disinfectants (chlorine, peracetic acid, hydrogen peroxide, ozone, etc.) or to other physical treatments (dry heat, UV-C, pulsed light, high hydrostatic pressure combined with temperature) to determine whether it would be lower, similar or higher than the resistance of group I and II *C. botulinum* strains.

Little work has also been done on the growth of *C. botulinum* group III in foodstuffs. Growth limits (temperature, NaCl, pH) for *C. botulinum* have been established for some strains. No growth at 10°C, growth of some strains at 12.8°C and above, and growth of all strains at 15.6°C have been reported (Segner et al., 1971). No growth was observed at salt concentrations above 3% NaCl, at pH 4.9 and below. In haddock, growth was found to be as rapid as that obtained in a laboratory culture medium in optimal growth conditions. In the absence of other specific data on growth or toxin production in foodstuffs, the nature of the media from which outbreaks of type C botulism originated suggests good adaptation of *C. botulinum* group III in many matrices containing food of animal origin. The temperature, pH or water activity that control the growth of group I strains (type A and B proteolytic) will most likely also control group III strains (Roberts and Gibson, 1979). In this context, growth tests or food challenges with surrogate microorganisms may be considered. Such surrogate strains of *C. botulinum* group I and group II have been proposed (Boix et al., 2022; Koukou et al., 2022; Poortmans et al., 2022). Data and models established with *C. sporogenes* as a surrogate of *C. botulinum* group I can help to define the control measures to prevent growth (Boix et al., 2022; Koukou et al., 2022). Unfortunately, no surrogate microorganism for *C. botulinum* group III has been defined yet.

Unfortunately, again, no data are available to suggest that the sensitivity of *C. botulinum* group III to other environmental factors present during food storage (redox, CO_2_-enriched modified atmosphere, etc.) or to preservatives is less than, similar to, or greater than the susceptibility of *C. botulinum* group I and II strains. In particular, the effect of nitrate and nitrite on *C. botulinum* group III, which are preservatives commonly applied for the control of *C. botulinum* groups I and II in meat processing and curing of meat or fish (Sofos et al., 1979; Skovgaard, 1992), is not documented to our knowledge, likely because of a very low association to human botulism, and thus an estimated unnecessity of specific control.

## 5. Conclusion

Botulism is still a major concern for animal and human health. The toxinotypes C, D, C/D, and D/C are far less known than A and B, especially regarding their potential impact on human health. The analysis of human botulism surveillance data worldwide over the period 1976–2018 confirms that the overwhelming majority of botulism forms are foodborne and infant botulism. The types of BoNTs involved are types A and B, then E, occasionally F. Over this period, no human cases of type C, C/D, D or D/C have been identified.

To address the issue of a potential zoonotic aspect of types C and D, we reviewed the existing literature on the rare cases reported worldwide since the 1950s (Meyer, 1953; Prévot et al., 1953; Demarchi et al., 1958; Fleming, 1960; Rey et al., 1964; Matveev et al., 1966; Maupas et al., 1976; Oguma et al., 1990; Martrenchar et al., 2019). The original articles were found and their reading seem to indicate a causal relationship between exposure to BoNT and/or *C. botulinum* type C and the occurrence of human botulism cases (two confirmed outbreaks). However, the sources of contamination have not been formally confirmed and there is still uncertainty about the zoonotic origin of these cases.

For type D, only one outbreak of foodborne botulism has been identified worldwide during the study period, for which exposure to type D BoNT was only suspected (Demarchi et al., 1958). The low sensitivity of humans to C, D and mosaic toxins is the preferred hypothesis to explain the almost total absence of case related to types C, D, C/D, and D/C. However, despite this low sensitivity of humans, spreading as fertilizer of manure or of agricultural waste known to be contaminated by *C. botulinum* and/or BoNT on crops of vegetables eaten raw should be avoided. Additionally, a thorough decontamination using biocides and more particularly sporicides (chlorine and hydrogen peroxide products being the most effective) must be implemented in farms affected by an outbreak of animal botulism. Land spreading of contaminated manure is not acceptable without a proper treatment of effluents (Anses, 2022). However, if the option of spreading the effluents from a botulism outbreak is chosen, the spreading equipment must limit emission of dust and aerosol. Effluent spreading must be performed under calm weather conditions and respect sufficient distances (at least 400 meters for effluents of poultry origin) to pastures and routes frequented by animals, to open cattle stabling, and dwellings and areas of human activity. Spreading on field crops is recommended and spreading on grasslands must be excluded. The vaccination of cattle in mixed farms and in any situation of potential exposure to animal botulism can be encouraged as a preventive measure. In addition to these recommendations, there is the need to wear a dust mask for operators carrying out the spreading.

As mentioned in this review, botulism occurs quite frequently in wild or farm birds and in bovine and involves types C, D, C/D, and D/C. It has also been reported with types C and D in other animal species such as horse (Swink and Gilsenan, 2022) and mink (Phaneuf et al., 1972; Wilson et al., 2015). However, these types of botulism are extremely rare in humans and implementation and maintenance of biosecurity measures could as well potentially contribute to keep these types of botulism extremely rare.

## Author contributions

FM coordinated and supervised the review work and contributed to writing and editing. FC, MFe, M-EF, MFo, PF, J-PG, LGr, LGu, DH, PK, SLB-L, CLM, CM, HM, KP, J-PV, and CW were all involved in the writing and editing process to a similar extent. All authors contributed to the article and approved the submitted version.

## Conflict of interest

The authors declare that the research was conducted in the absence of any commercial or financial relationships that could be construed as a potential conflict of interest.

## Publisher’s note

All claims expressed in this article are solely those of the authors and do not necessarily represent those of their affiliated organizations, or those of the publisher, the editors and the reviewers. Any product that may be evaluated in this article, or claim that may be made by its manufacturer, is not guaranteed or endorsed by the publisher.
